# Middle-aged predominance and diagnostic delays in anti-LGI1 encephalitis: the role of antibody testing

**DOI:** 10.3389/fneur.2026.1777478

**Published:** 2026-05-14

**Authors:** Shu Kan, Gege Zhang, Hua Yu, Hongmei Ding

**Affiliations:** 1 Department of Neurology, The Affiliated Hospital of Xuzhou Medical University, Xuzhou, China; 2 Xuzhou Medical University, Xuzhou, China; 3 Department of Pediatrics, Xuzhou Municipal First People’s Hospital, Xuzhou, China

**Keywords:** autoimmune encephalitis, faciobrachial dystonic seizures, hyponatremia, leucine-rich glioma-inactivated 1, prognosis

## Abstract

**Background:**

This study aimed to investigate the clinical characteristics of anti-leucine-rich glioma-inactivated 1 (LGI1) antibody encephalitis and to identify significant risk factors associated with long-term functional prognosis.

**Methods:**

We retrospectively analyzed the clinical data of 20 patients diagnosed with anti-LGI1 encephalitis at the Affiliated Hospital of Xuzhou Medical University from January 2021 to October 2024. All patients underwent a 12-month follow-up. Functional outcomes were assessed using the modified Rankin Scale (mRS) at 12 months post-discharge and were dichotomized into favorable (mRS ≤ 2) and poor (mRS > 2) outcome groups.

**Results:**

Of the 20 patients (14 males and 6 females; mean age: 57 years), seizures represented the most prevalent chief complaint at onset (70%). Cognitive impairment was noted as an initial presenting symptom in 45% of patients, whereas its cumulative incidence reached 95% throughout the entire disease course. Pathognomonic faciobrachial dystonic seizures (FBDS) occurred in 40% of cases, and hyponatremia was present in 70% of the cohort. Anti-LGI1 antibodies were detected in 95% (19/20) of serum samples and 90% (18/20) of cerebrospinal fluid (CSF) samples. Notably, 40% of patients exhibited unremarkable cranial MRI findings, and 55% demonstrated normal CSF protein levels upon admission. At the 12-month follow-up, 80% of patients achieved favorable functional recovery. Univariate analysis revealed that advanced age (*p* = 0.006), prolonged diagnostic delay (*p* = 0.009), and higher antibody titers in both serum (*p* = 0.005) and CSF (*p* = 0.009) were significantly correlated with poor functional outcomes.

**Conclusion:**

The high frequency of unremarkable results in initial ancillary investigations, such as MRI and routine CSF analysis, often leads to substantial diagnostic and therapeutic delays in anti-LGI1 encephalitis. Advanced age, diagnostic latency, and high antibody titers are critical predictors of poor prognosis. Early and simultaneous antibody screening in both serum and CSF is essential for narrowing the diagnostic window and optimizing long-term neurological recovery.

## 1 Introduction

Autoimmune encephalitis (AE) comprises a group of inflammatory disorders of the central nervous system characterized by antibodies targeting neuronal surface proteins. (1) Among these, anti-LGI1 encephalitis typically manifests as limbic encephalitis (2). Current literature has established faciobrachial dystonic seizures (FBDS) and hyponatremia as diagnostic hallmarks of the disease (3, 4). However, a significant clinical paradox remains: a substantial proportion of patients exhibit unremarkable findings in conventional MRI and routine CSF analysis during early stages, frequently leading to misdiagnosis.

While recent studies, such as Chen et al. (5), have comprehensively described general clinical and imaging features, the specific prognostic value of quantifying antibody titers simultaneously in both serum and cerebrospinal fluid (CSF) remains insufficiently characterized. Most existing reports focus on broad clinical descriptions rather than the quantitative relationship between multi-compartment antibody levels and long-term functional recovery.

Distinguishing our work from these broader descriptions, this study focuses on the synergistic value of dual serum and CSF antibody testing. By specifically quantifying the influence of diagnostic latency and multi-compartment antibody titers on 12-month outcomes, we aim to provide clinicians with more precise prognostic indicators to optimize the therapeutic window and prevent irreversible neurological sequelae.

## 2 Methods

### 2.1 Patient selection

**Figure 1:**
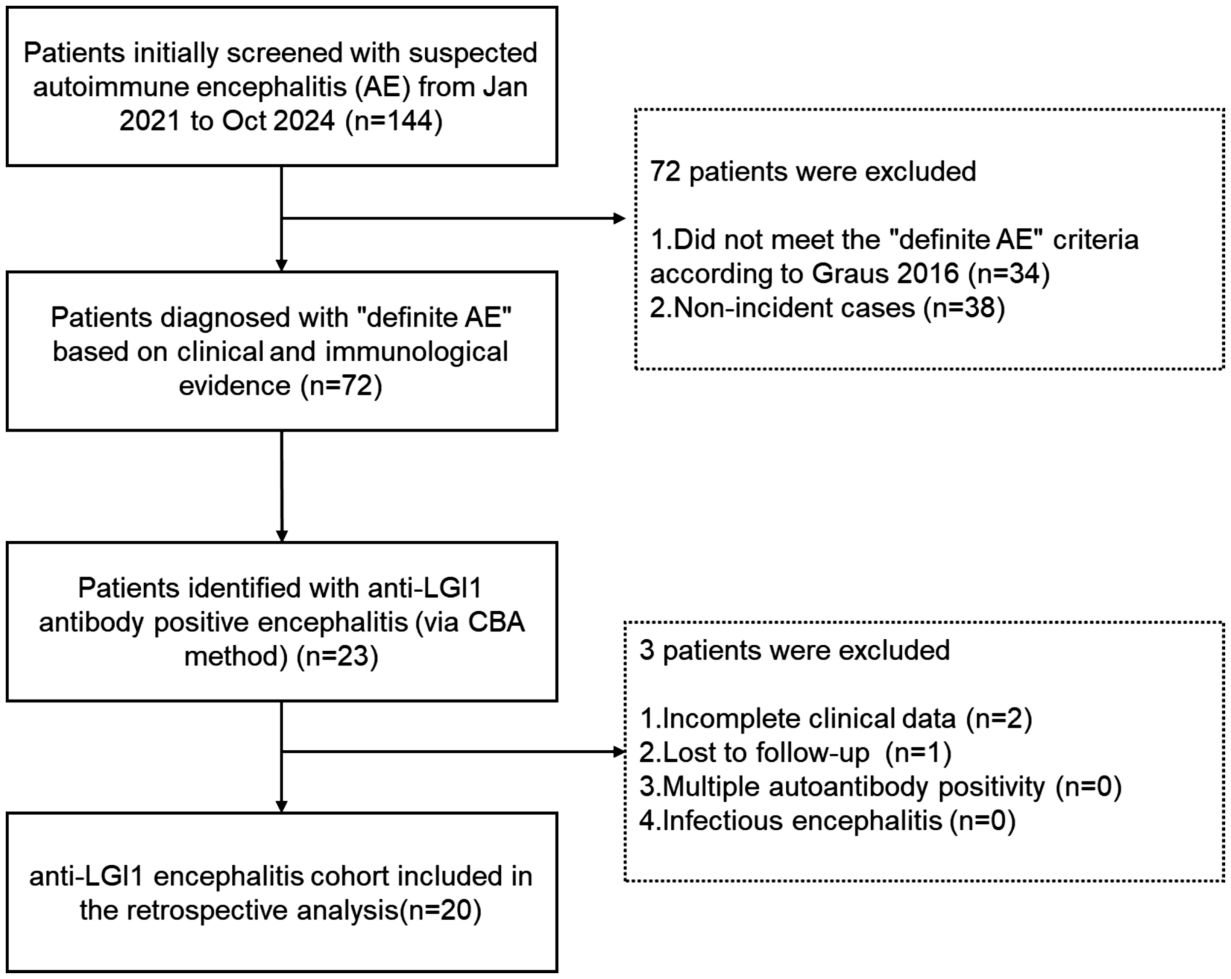
The flowchart of patient selection.

A retrospective analysis was conducted on patients with suspected AE at the Affiliated Hospital of Xuzhou Medical University between January 1, 2021, and October 1, 2024. As illustrated in the flowchart (Figure 1), a total of 144 patients were initially screened. The diagnosis of anti-LGI1 encephalitis was established according to the criteria adapted from Graus et al. (6): (1) acute or subacute onset of symptoms suggestive of limbic system involvement (e.g., memory deficits, seizures, or psychiatric symptoms); (2) standardized detection of LGI1-IgG antibodies in serum and/or CSF using cell-based assays (CBA); and (3) exclusion of alternative etiologies.

To minimize selection bias and ensure diagnostic accuracy, all candidates underwent a comprehensive diagnostic workup to rule out other etiologies. Infectious encephalitis was excluded through CSF metagenomic Next-Generation Sequencing (mNGS), Gram staining, and cultures. To evaluate potential paraneoplastic etiologies and occult malignancies, patients underwent systemic screening, including serum tumor marker assays, paraneoplastic antibody panels, and computed tomography (CT) of the chest, abdomen, and pelvis. Cranial MRI and EEG were routinely performed for all patients. Patients were excluded if they had incomplete clinical data, were lost to follow-up, or demonstrated multiple neuronal autoantibody positivity. Ultimately, 20 patients were included in the final analysis.

### 2.2 Clinical assessment and ethics statement

Retrospective clinical data, including demographics, chief complaints, laboratory findings, and therapeutic regimens, were extracted. Disease severity at admission was evaluated using the Clinical Assessment Scale in Autoimmune Encephalitis (CASE) (7). Standardized brain MRI and CSF analysis were completed within 48 h of hospitalization to ensure consistency in baseline evaluation.

Functional outcomes at 12 months post-discharge were assessed using the modified Rankin Scale (mRS). Although the functional status reflects a 12-month recovery period, these data were retrospectively extracted from outpatient records or standard-of-care telephone follow-ups conducted by neurologists blinded to the initial diagnostic latency. Patients were dichotomized into favorable (mRS ≤ 2) and poor (mRS > 2) outcome groups. The study protocol was approved by the Ethics Committee of the Affiliated Hospital of Xuzhou Medical University (Approval No. [XYFY-2025-KL469-01]). In accordance with national regulations, the requirement for written informed consent was waived due to the study’s retrospective, non-interventional design based on pre-existing clinical records.

### 2.3 Statistical analysis

Statistical analyses were performed using R software. Continuous variables were expressed as mean ± standard deviation (SD) or median (interquartile range, IQR) and compared using the Student’s *t*-test or Mann–Whitney *U* test. Categorical variables were presented as frequencies (%) and compared using the chi-square test or Fisher’s exact test. Spearman’s correlation analysis was conducted to evaluate the associations between clinical variables (age, diagnostic latency, and antibody titers) and 12-month mRS scores. A two-sided *p*-value < 0.05 was considered statistically significant. Given the restricted sample size (*N* = 20) and potential multicollinearity between serum and CSF antibody titers, multivariate regression analysis was not performed to avoid model overfitting.

A flowchart detailing the patient screening and enrollment process is presented in Figure 1. Briefly, a total of 144 patients with a suspected or confirmed diagnosis of AE were screened at the Affiliated Hospital of Xuzhou Medical University between January 1, 2021, and October 1, 2024.

## 3 Results

### 3.1 Patient characteristics

Upon admission, all patients underwent a standardized diagnostic evaluation. Brain MRI and CSF analysis were completed within 48 h of hospitalization. The median interval from symptom onset to these procedures was 25 days (8,94). Twenty patients (14 males, 6 females) meeting the diagnostic criteria for anti-LGI1 encephalitis were retrospectively enrolled. Their baseline characteristics are summarized in Table 1. Notably, 40% of the patients (*n* = 8) had been initially misdiagnosed at other institutions. Seizures were the most common chief complaint (70%, 14/20), with FBDS observed in 57.1% (8/14) of these cases. Cognitive impairment was the second most frequent initial manifestation, occurring as a chief complaint in 45% (9/20) of cases. Psychiatric disturbances, including emotional instability, behavioral changes, or personality shifts, were identified in 85% (17/20) of the patients. Furthermore, autonomic dysfunction, characterized by symptoms such as cardiovascular instability or sudomotor abnormalities, was documented in 40% (8/20) of the cohort. These non-motor features frequently co-existed with cognitive impairment and seizures, highlighting the complex multisystem involvement of anti-LGI1 encephalitis. However, when evaluating the total disease course, the cumulative incidence of cognitive impairment reached 95% (19/20), as detailed in Table 1. Hyponatremia was present in 70% (14/20) of the cohort. Regarding diagnostic workups, 90% (18/20) of patients had normal CSF WBC counts, while 55% (11/20) exhibited normal protein levels. Unremarkable brain MRI findings were observed in 40% (8/20) of cases. Notably, 3 patients demonstrated both normal CSF parameters (cell count and protein) and unremarkable MRI findings. The mean CSF opening pressure was 150 ± 31.21 mm H₂O. Anti-LGI1 antibodies were undetectable in the serum of one patient (CSF titer 1:1), whereas two patients showed negative CSF antibodies despite positive serum titers (1:10). All patients received first-line immunotherapy (intravenous immunoglobulin and /or corticosteroids), and 15% (3/20) required second-line agents, including mycophenolate mofetil (MMF), azathioprine (AZA), rituximab (RTX), or cyclophosphamide (CTX). At the 12-month follow-up, four patients (20.0%) had poor functional outcomes (mRS > 2), including one death.

**Table 1:** Demographic and clinical characteristics of 20 patients with anti-LGI1 encephalitis.

Pt. No.	Gender	Age (Y)	Initial visit	Time to diagnosis (day)	Chief Complaint at Onset	Cumulative Symptoms (Total Course)	CSF open pressure (mmH2O)	CSF WBC count (×10^6^)	CSF protein (g/l)	Serum sodium (mmol/L)	Cranial MRI	EEG	Baseline CASE	Therapeutic regimen	CSF antibody titers	Serum antibody titer	MRS
1	M	56	Y	30	Sz/FBDS	CD/FBDS/PD/Sz	185	−	−	135.5	+	+	4	GC/IVIG	1:10	1:100	1
2	F	60	N	365	CD	AD/CD/PD	170	−	−	137.0	+	+	3	GC/IVIG	1:10	1:320	2
3	M	54	Y	14	CD	AD/CD/PD	160	24	−	135.8	+	−	3	GC/IVIG	1:10	1:1000	2
4	M	58	N	120	CD	CD/PD	140	−	−	136.4	+	+	3	GC/IVIG	1:1	1:100	1
5	M	64	N	60	CD/Sz	AD/CD/PD/Sz	220	−	−	129.2	+	+	6	GC/IVIG/MMF	1:10	1:32	1
6	M	54	N	60	FBDS	CD/FBDS/PD	155	−	0.68	128.2	+	−	3	GC/AZA/RTX/CTX	1:3.2	1:32	2
7	M	83	N	120	FBDS/Sz /CD	CD/FBDS/PD/Sz	100	−	0.85	122.7	−	+	12	GC/IVIG	1:100	1:1000	6
8	F	64	Y	3	Sz	CD/Sz	185	−	1.23	122.9	+	−	2	GC/IVIG	1:10	1:100	0
9	M	25	Y	7	FBDS/CD	CD/FBDS/PD	165	−	−	136.3	−	−	4	GC/IVIG	1:1	1:32	0
10	M	76	Y	20	Dz	CD/Dz	85	−	−	136	−	+	2	GC	1:1	1:100	1
11	M	73	Y	140	FBDS	AD/CD/Dz/FBDS/PD	150	−	0.82	126.5	−	−	2	GC/IVIG	1:10	1:1000	3
12	M	61	Y	6	FBDS	CD/FBDS/PD	150	−	0.82	127.4	−	−	2	GC	1:1	1:32	0
13	M	27	Y	8	FBDS	CD/FBDS/PD	160	−	−	139.6	+	−	2	GC/IVIG	1:1	1:100	0
14	M	76	N	730	Sz	AD/CD/PD/Sz	140	−	−	144.4	+	+	3	GC/IVIG	1:10	1:320	3
15	F	62	Y	10	CD	AD/CD/PD	105	−	1.1	142.1	−	+	3	GC/IVIG	1:10	1:10	1
16	M	60	N	60	Sz	AD/CD/Sz	140	−	−	140.5	−	−	2	GC/IVIG	1:1	1:100	2
17	F	26	Y	7	Sz	AD/CD/PD/Sz	150	−	−	133.4	+	+	3	GC/IVIG	−	1:10	1
18	F	24	N	8	Dz	Dz/PD	169	78	1.3	139.8	+	−	3	GC/IVIG	−	1:10	0
19	F	53	Y	15	Sz/CD	CD/PD/Sz	150	−	0.51	136.8	+	+	4	GC/IVIG	1:1	−	1
20	M	80	Y	68	FBDS/CD	CD/FBDS/PD	120	−	0.65	134.5	−	−	19	GC/AZA	1:100	1:1000	3

Individual patient data are presented. For categorical variables, “Y” indicates presence and “N” indicates absence; for laboratory and imaging results, “(+)” indicates abnormal/positive findings, and “(−)” indicates normal/negative findings.AD, autonomic dysfunction; AZA, azathioprine; CD, cognitive disorder; CSF, cerebrospinal fluid; CTX, cyclophosphamide; Dz, dizziness; EEG, electroencephalogram; FBDS, faciobrachial dystonic seizures; GC, glucocorticoids; IVIG, intravenous immunoglobulin; LGI1, leucine-rich glioma-inactivated 1; MMF, mycophenolate mofetil; MRI, magnetic resonance imaging; mRS, modified Rankin Scale; PD, psychiatric disorders; Pt. No., patient number; RTX, rituximab; Sz, seizure; WBC, white blood cell.

The clinical characteristics of the 20 patients are summarized in Table 1.

### 3.2 Univariate analysis of predictors of prognosis

Four patients (20.0%) experienced poor functional outcomes (mRS > 2), including one mortality. Older age was significantly associated with a poorer prognosis (78.0 ± 4.4 VS 51.5 ± 16.4 years; *p* = 0.006). Notably, patients in the poor-prognosis group exhibited significantly higher anti-LGI1 antibody titers in both serum and CSF compared to those with favorable outcomes (*p* < 0.01 and *p* < 0.05, respectively). In contrast, no significant differences were observed between the two groups regarding routine CSF parameters (white cell count and biochemical markers), sex, serum sodium levels, or EEG and MRI findings (*p* > 0.05; Table 2). Furthermore, the time to diagnosis was significantly longer in the poor-outcome group compared to the good-outcome group [median 130 (81, 582) days vs. 14 (7, 60) days; *p* = 0.009].

**Table 2:** Comparison of clinical and laboratory characteristics between patients with favorable and poor outcomes.

Characteristics	Good prognosis	Poor prognosis	z/t/X^2^	*p*-value
Age	51.5 ± 16.4	78 ± 4.4	−3.140	0.006
Sex Male	10	4		0.267
Female	6	0		
Time to diagnosis (days)	14(7,60)	130(81,582)	−2.605	0.009
Serum sodium (mmol/L)	134.8 ± 5.3	132.0 ± 9.6	0.798	0.436
EEG Normal	8	2		1.000
Abnormal	8	2		
CSF opening pressure (mmH_2_O)	155.6 ± 31.1	127.5 ± 22.2	1.684	0.109
CSF WBC count normal	14	4		1.0
Abnormal	2	0		
CSF protein normal	10	1		0.285
Abnormal	6	3		
Cranial MRI normal	5	3		0.255
abnormal	11	1		
Baseline CASE	3(2,3.75)	7.5(2.25,17.25)	−1.091	0.275
Serum antibody titers			−2.804	0.005
Negative	1	0		
1:10	3	0		
1:32	4	0		
1:100	6	0		
1:320	1	1		
1:1000	1	3		
CSF antibody titers			−2.599	0.009
Negative	2	0		
1:1	7	0		
1:3.2	1	0		
1:10	6	2		
1:32	0	0		
1:100	0	2		
Second-line treatment Yes	2	1		0.509
No	14	3		

Data are expressed as mean ± SD, median (range), or n (%). Statistical significance was determined using the [Student’s *t*-test, Mann–Whitney U test, or Fisher’s exact test]. *p* < 0.05 was considered statistically significant.

**Figure 2:**
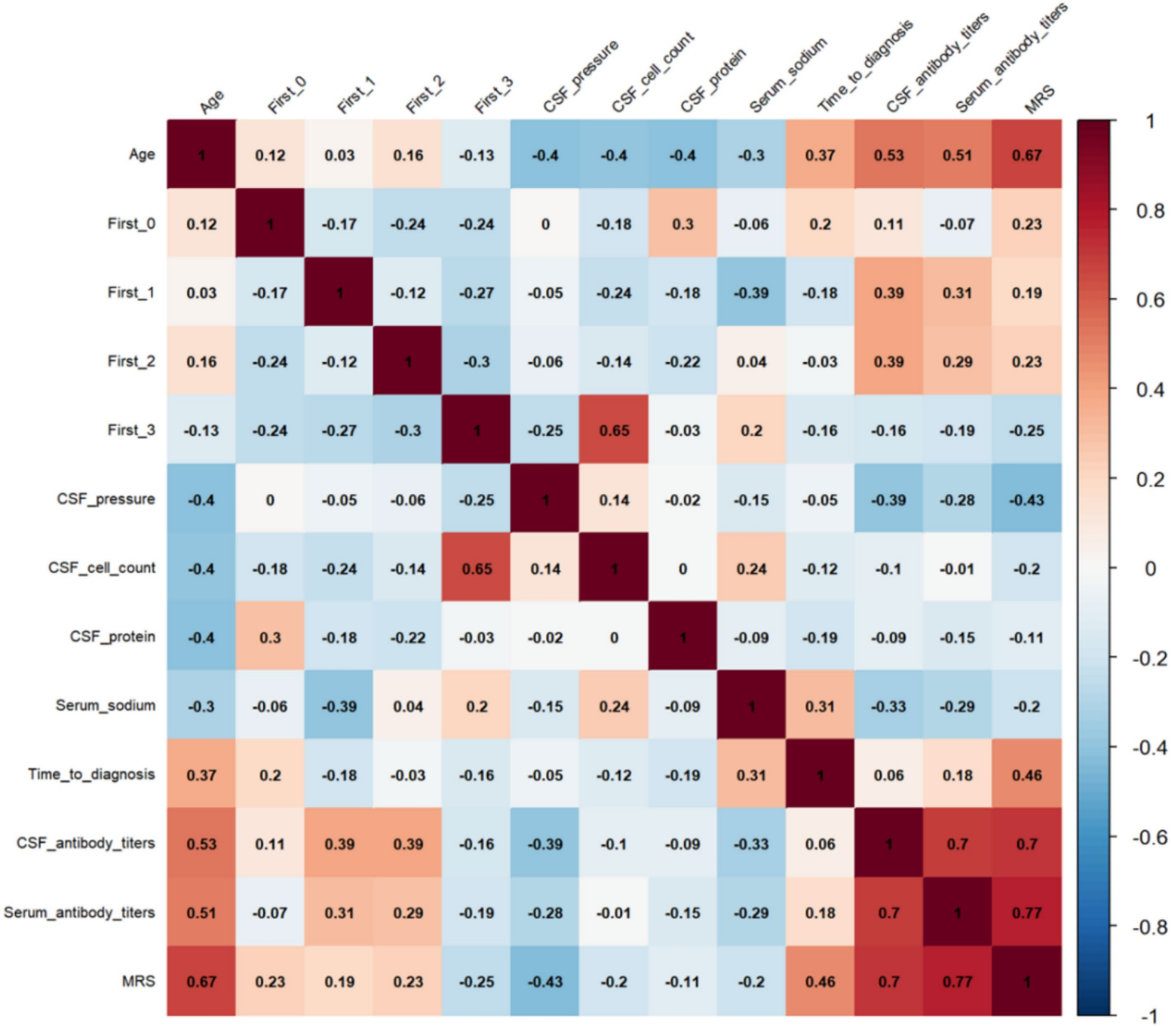
Correlation analysis of 13 variables and functional outcomes. First 0: Seizures First1: FBDS First2: Cognitive disorder First3 Dizziness.

Correlation analysis of 13 clinical variables was performed, as illustrated in the heatmap (Figure 2). Age, CSF antibody titers, serum antibody titers, and Time to diagnosis all exhibited significant positive correlations with 12-month mRS scores (*p* < 0.05). Among these, serum antibody titer demonstrated the strongest association with functional disability (r = 0.77, *p* < 0.001). No significant correlations were found for other variables, including seizures, FBDS, cognitive disorder, or dizziness.

## 4 Discussion

In this retrospective study, we systematically evaluated the clinical and epidemiological profiles of patients with anti-LGI1 encephalitis, revealing several key prognostic indicators. As the second most prevalent form of AE (8), anti-LGI1 encephalitis is characterized by its disruption of the LGI1 protein, which orchestrates neuronal excitability and synaptic strength via Kv1.1 channels and AMPA receptors (9). Crucially, our findings demonstrate that serum antibody positivity exhibits a stronger correlation with clinical prognosis than CSF results, underscoring the indispensable role of comprehensive antibody screening in the diagnostic workup. Furthermore, we identified that advanced age is a significant risk factor for poor outcomes, suggesting that elderly patients require more vigilant monitoring and potentially more aggressive intervention. Given the diagnostic challenges posed by its low incidence and often unremarkable early-stage ancillary findings (10), these insights into antibody status and age-related prognosis are vital for facilitating early recognition and optimizing long-term neurological recovery.

In our cohort of 20 patients with anti-LGI1 encephalitis, we observed a distinct male predominance (70% vs. 30%) and a median age of 60 years. These demographic findings reinforce the prevailing consensus that this subtype of AE predominantly affects middle-aged and elderly men, aligning with the observations of Fels et al. and Irani et al. (11, 12). Clinically, seizures, FBDS, and cognitive impairment were the most prevalent manifestations. These symptoms likely stem from the disruption of LGI1’s physiological function as a critical component of the voltage-gated potassium channel (VGKC) complex. Specifically, the loss of LGI1 function enhances intrinsic cellular excitability and facilitates glutamatergic synaptic transmission within hippocampal CA3 neurons, providing a mechanistic basis for the observed clinical phenomena (13).

Previous literature indicates that approximately 20 to 40% of patients with anti-LGI1 encephalitis experience FBDS, predominantly middle-aged men. Clinically, these seizures present as brief, paroxysmal, and stereotyped movements. The classic semiology involves unilateral arm flexion, ipsilateral facial twitching, and involuntary hand opening that frequently causes patients to drop objects. Occasionally, the lower extremities may also be affected (14). Current evidence suggests that FBDS originates at the basal ganglia level and is triggered by focal cortical discharges, involving a fronto-temporo-basal ganglia loop (15). Consequently, FBDS may precede or follow the onset of temporal lobe seizures. In our cohort, 8 patients (40%) presented with FBDS, reinforcing its status as a pathognomonic marker for anti-LGI1 encephalitis. Early recognition of these pivotal indicators is crucial for timely intervention to prevent further clinical deterioration.

Regarding cognitive impairment, our study revealed a striking discrepancy between disease onset and the total disease course. While cognitive decline was reported as the primary chief complaint in only 45% of patients at onset, its cumulative incidence ultimately reached 95%. This cumulative rate aligns well with previous large-scale cohorts which demonstrate that cognitive deficits are a nearly universal feature of anti-LGI1 encephalitis (3). The relatively lower frequency of cognitive complaints at initial presentation may be attributed to the prominence of more acute and visually dramatic symptoms, such as FBDS or generalized seizures. These acute events often prompt urgent medical evaluation, whereas early-stage subtle memory loss might be overlooked or misattributed to normal aging by caregivers. This underscores the necessity for comprehensive cognitive assessments in middle-aged and elderly patients presenting with new-onset seizure.

Furthermore, hyponatremia was observed in 70% of our patients, serving as another hallmark of this condition. While the underlying mechanism remains partially elusive, several hypotheses have been proposed. Some studies suggest that psychogenic polydipsia—leading to excessive fluid intake—may be a primary driver, though it remains unclear whether this intense thirst stems from an organic hypothalamic–pituitary axis inflammation or a secondary delusional phenomenon (16). Alternatively, since LGI1 is expressed within the renal tubular system, sodium homeostasis may be directly disrupted. Another compelling mechanism involves LGI1 antibody-mediated binding to ADH-producing neurons in the hypothalamic paraventricular nucleus, subsequently inducing water retention (17, 18).

A pivotal finding of this study is the robust correlation between diagnostic delay and poor functional outcomes (*p* = 0.009), highlighting a critical “therapeutic window” where protracted intervention lags may lead to irreversible neuronal damage. In our cohort, many patients experienced a “diagnostic odyssey”—with delays exceeding 1 year in several instances—primarily due to the insidious onset of symptoms and the frequently unremarkable results of routine ancillary investigations. Specifically, 40% of our patients presented with unremarkable brain MRI findings, and 55% demonstrated normal routine CSF parameters, a pattern that diverges significantly from conventional viral or other AE. Such non-inflammatory profiles often lead to misdiagnoses of primary epilepsy or neurodegenerative cognitive disorders in community settings, bridging the gap to clinical deterioration.

The prevalence of these “normal” routine findings may be rooted in the unique immunological profile of anti-LGI1 encephalitis. Unlike anti-NMDAR encephalitis, which involves pro-inflammatory IgG1 antibodies and frequent CSF pleocytosis, anti-LGI1 antibodies predominantly belong to the IgG4 subclass (12, 15, 19). This subclass typically does not robustly activate the complement cascade, resulting in a less pronounced inflammatory response in the CSF. Furthermore, our data reinforce the observation that LGI1 antibodies exhibit substantially higher sensitivity in serum than in CSF, with serum titers often being 10 to 100 times more concentrated (20). This explains the “serum-positive but CSF-negative” profile observed in a subset of our patients and underscores the limitation of relying solely on CSF analysis. Therefore, to minimize the risk of underdiagnosis and mitigate the consequences of diagnostic delay, we strongly recommend simultaneous antibody screening in both serum and CSF. For middle-aged and elderly patients presenting with subacute cognitive decline or FBDS, early serological profiling is indispensable to facilitate prompt immunotherapy and optimize long-term prognosis.

Although anti-LGI1 encephalitis generally carries a more favorable prognosis compared to anti-NMDAR encephalitis (21), approximately 20% of our cohort experienced poor functional outcomes, including one fatality (5%) following refractory treatment. Consistent with broader observations in AE (22), our study identifies advanced age as a significant predictor of poorer prognosis. This vulnerability in elderly patients may stem from the increased complexity of geriatric comorbidities and diminished physiological resilience. Furthermore, we observed that the interval to accurate diagnosis was significantly longer in the poor prognosis group compared to those with favorable outcomes (*p* < 0.05), echoing the premise that delayed initiation of immunotherapy is a primary driver of treatment resistance in adults (23).

Untreated autoimmune limbic encephalitis carries the risk of progressing into frontotemporal dementia, severely compromising long-term quality of life (24). Given that early MRI and routine CSF investigations frequently yield unremarkable results, antibody testing emerges as a paramount diagnostic tool. In our analysis, key adverse prognostic factors included diagnostic delay, advanced age, and high antibody titers, with the latter correlating with clinical severity—a finding aligned with Cui et al. (25). Collectively, these findings reinforce the imperative for early, comprehensive antibody evaluation to facilitate timely intervention and mitigate irreversible neurological decline.

Furthermore, our analysis revealed that treatment intensity did not significantly differ between the two prognostic groups (Table 2), with both cohorts achieving high rates of first-line therapy coverage. The observation that a subset of patients experienced poor outcomes despite receiving treatment regimens comparable to those with favorable outcomes underscores the critical influence of non-therapeutic factors. Advanced age and prolonged diagnostic delays likely cause permanent neuronal injury. Once these neurological deficits are established, late-stage immunotherapy often fails to fully reverse them. These findings suggest that the clinical outcome may be more heavily predicated on early intervention than on the escalation of treatment intensity once permanent damage has occurred.

Despite the insights provided, several limitations of this study warrant consideration. First, the relatively small sample size (*N* = 20) may constrain the statistical power and the generalizability of our findings. However, it is noteworthy that anti-LGI1 encephalitis is an orphan disease with an exceptionally low estimated annual incidence of approximately 0.83 per million (26). Our cohort, consisting of a consecutive series of cases from a high-volume tertiary center, ensures a highly standardized diagnostic and therapeutic approach. Furthermore, the restricted sample size precluded the performance of a multivariate regression analysis. Given the inherent recruitment challenges associated with such rare conditions, well-characterized cohorts are essential to provide foundational clinical insights. Nonetheless, future multi-center prospective studies with larger populations are necessary to validate these results and establish more robust prognostic models.

Second, the retrospective nature of the study may introduce potential information bias. Third, FDG PET/CT was not performed in our cohort due to limited accessibility. Previous literature has demonstrated that PET/CT can reveal pathognomonic metabolic abnormalities, such as bilateral basal ganglia hypermetabolism, which often precede structural MRI changes in anti-LGI1 encephalitis (27). The absence of functional imaging data may have limited our capacity to detect early-stage cerebral involvement in MRI-negative cases. Future investigations incorporating multi-modal neuroimaging could further refine our understanding of the early pathogenic phase.

## 5 Conclusion

Our study demonstrates that anti-LGI1 encephalitis predominantly affects middle-aged and elderly individuals, with a peak incidence observed between 55 and 60 years. FBDS and hyponatremia represent highly specific clinical hallmarks. Diagnostic challenges remain significant, as conventional investigations—including structural MRI, routine CSF analysis, and electroencephalography—frequently yield unremarkable results, often culminating in misdiagnosis or therapeutic delays. Conversely, the detection of specific antibodies in both serum and CSF provides substantial diagnostic and prognostic value. Early clinical suspicion, followed by comprehensive serological and intrathecal antibody profiling, is indispensable for facilitating prompt intervention and optimizing long-term functional recovery.

## Data Availability

The original contributions presented in the study are included in the article/supplementary material, further inquiries can be directed to the corresponding author/s.
